# Simultaneous model discrimination and parameter estimation in dynamic models of cellular systems

**DOI:** 10.1186/1752-0509-7-76

**Published:** 2013-08-12

**Authors:** Maria Rodriguez-Fernandez, Markus Rehberg, Andreas Kremling, Julio R Banga

**Affiliations:** 1 Institute for Collaborative Biotechnologies, University of California, Santa Barbara, CA 93106-5080, USA; 2 Max Planck Institute for Dynamics of Complex Technical Systems, Sandtorstr. 1, 39106 Magdeburg, Germany; 3 Faculty of Mechanical Engineering Specialty Division for Systems Biotechnology, Technische Universitat München, Boltzmannstr. 15, 85748 Garching, Germany; 4 (Bio) Process Engineering Group, IIM-CSIC, C/Eduardo Cabello 6, 36208 Vigo, Spain

**Keywords:** Dynamic modelling, Parameter estimation, Model discrimination, Global optimization

## Abstract

**Background:**

Model development is a key task in systems biology, which typically starts from an initial model candidate and, involving an iterative cycle of hypotheses-driven model modifications, leads to new experimentation and subsequent model identification steps. The final product of this cycle is a satisfactory refined model of the biological phenomena under study. During such iterative model development, researchers frequently propose a set of model candidates from which the best alternative must be selected. Here we consider this problem of model selection and formulate it as a simultaneous model selection and parameter identification problem. More precisely, we consider a general mixed-integer nonlinear programming (MINLP) formulation for model selection and identification, with emphasis on dynamic models consisting of sets of either ODEs (ordinary differential equations) or DAEs (differential algebraic equations).

**Results:**

We solved the MINLP formulation for model selection and identification using an algorithm based on *Scatter Search* (SS). We illustrate the capabilities and efficiency of the proposed strategy with a case study considering the KdpD/KdpE system regulating potassium homeostasis in *Escherichia coli*. The proposed approach resulted in a final model that presents a better fit to the *in silico* generated experimental data.

**Conclusions:**

The presented MINLP-based optimization approach for nested-model selection and identification is a powerful methodology for model development in systems biology. This strategy can be used to perform model selection and parameter estimation in one single step, thus greatly reducing the number of experiments and computations of traditional modeling approaches.

## Background

Model development is a key task in systems biology, and involves different steps, such as model calibration, experimental design and model refinement which usually take place in an iterative way (see reviews in [1-5]). The process of building a model of a biological system typically starts by generating an initial model candidate, or by taking one from the pre-existing knowledge, and then involves an iterative cycle of hypotheses-driven model modifications, new experimentation and subsequent model identification steps, finally leading to a satisfactory refined model [6,7]. Thus, model selection, experimentation and model refinement can be considered the basic elements of systems biology [8].

A number of researches have proposed different iterative schemes for model development involving the steps of parameter estimation, identifiability analysis, and optimal experimental design [9-12]. The related topic of optimal experimental design for parameter estimation [3,13] and for model discrimination [14-16] is receiving increased attention in recent years. Lillacci and Khammash [17] introduced a new method for parameter estimation based on Kalman filtering that can also be used to discriminate among alternate models of the same biological process.

Verheijen [18] presented an overview of model selection practices, highlighting the main criteria for choosing out of a large set of models: level of rigor, accuracy with respect to data, adequacy of the model, and its flexibility and computational complexity. He also identifies developments in optimization-based approaches [19,20] as very promising, but recognizing its limitations due to numerical and algorithmic challenges. Although research along this line has continued [21,22], it still remains as a challenging numerical problem.

Here, we present a method to simultaneously select a model and calibrate it in a single step. This contribution is based on the following four key ideas: (i) frequently, iterative model development cycles can be considered in a more compact way if sets of hypotheses can be grouped together and formulated as a parameterized set of models, from which the best alternative must be selected; (ii) we consider the problem of model selection formulating it as a simultaneous model selection and parameter identification problem; (iii) further, in order to make the selection decision in a systematic way, we formulate it as an optimization problem [23] acting on the parameterized set of models; (iv) the optimization problem, which belongs to the class of mixed-integer nonlinear programming (MINLP) problems, is solved by recently developed algorithms based on metaheuristics.

The paper is organized as follows: First, we describe the framework used for model selection and identification, based on the nested models paradigm. Then we state the corresponding optimization problem using a formulation based on mixed-integer non-linear programming subject to differential and algebraic constraints. In the following sections, we describe the application of this methodology to a case study considering a dynamic model of the KdpD/KdpE system of *Escherichia coli*. Finally, we provide a discussion of the results and summarize the main conclusions of the study.

## Methods

To the best of our knowledge, this is the first time that an MINLP framework for simultaneous model selection and identification is presented. The key issues for the successful design of this combined approach are: (i) selection of the integer and binary parameters that accurately describe all the possible nested models; (ii) reliable and accurate parameter estimation; (iii) use of efficient algorithms with reduced computational cost; (iv) assessment of model identifiability.

### Nested-models: selection and identification

**Figure 1:**
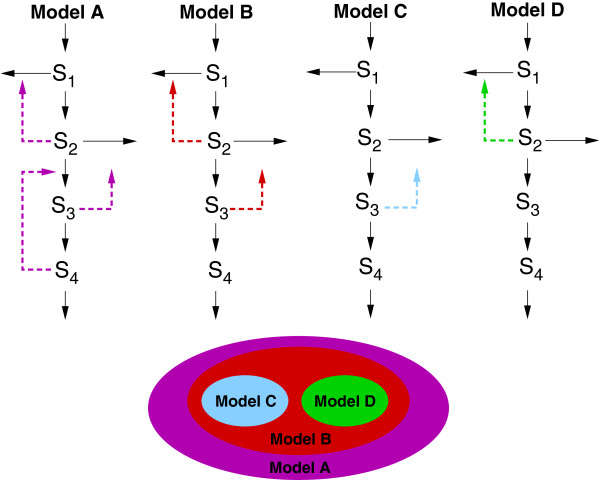
**Example of nested models.** Example of nested models where Model C and Model D are nested within Model B that is in turn nested within Model A, which can be considered as a superstructure.

In this contribution we consider dynamic models which are nested, i.e. there is a hierarchy such that each model is a particular subcase of an extended parameterized model, which can be considered as a superstructure. These nested-models arise from existing models plus new hypotheses such as e.g. the existence of new positive or negative feedback loops. In a loose sense, we can say that Model B is nested within Model A if Model B is a special case of Model A. Figure 1 depicts an example where Model A is a superstructure containing three feedback loops, Model B contains only two of them, and Model C and Model D one each. Therefore, we can say that Model C and Model D are nested within Model B that is in turn nested within Model A. In this framework, the model selection problem reads as follows: given a set of nested models, find the one which gives the best fit to the available experimental data, meeting possible additional constraints on model rigor, accuracy and adequacy.

Several functions have been suggested as metrics to asses the goodness of models fit. The maximum-likelihood estimation (MLE), introduced by Fisher in 1912 [24], consists of maximizing the so-called likelihood function which is the probability density of a model for the occurrence of the measurements for given parameters. Assuming the probability of the measurements to be uncorrelated normal distributions, the log-likelihood function (*J*_*ml*_, which yields to the same estimate than the likelihood function but is easier to handle in practice) is given as:

(1) $$\begin{array}{c} J_{\text{ml}}(\text{p})=\text{ln}\left(\overset{\text{NE}}{\underset{i=1}{\prod }}\overset{{\text{NV}}_{i}}{\underset{j=1}{\prod }}\overset{{\text{NM}}_{\text{ij}}}{\underset{k=1}{\prod }}{\left(\frac{1}{2\pi \sigma_{\text{ijk}}^{2}}\right)}^{\frac{1}{2}}\right)\\ \;-\frac{1}{2}\left\{\overset{\text{NE}}{\underset{i=1}{\sum }}\overset{{\text{NV}}_{i}}{\underset{j=1}{\sum }}\overset{{\text{NM}}_{\text{ij}}}{\underset{k=1}{\sum }}\right)\left(\left[\frac{{\left({\tilde{y}}_{\text{ijk}}-y_{\text{ijk}}(\text{p})\right)}^{2}}{\sigma_{\text{ijk}}^{2}}\right]\right\} \end{array}$$

where **p**: set of parameters to be estimated *NE*: number of experiments *NV*_*i*_: number of measured variables in experiment *iNM*_*ij*_: number of measurements of the variable *j* in experiment *i*$\sigma_{\text{ijk}}^{2}$: variance of the measurement *k* of the variable *j* in experiment *i*${\tilde{y}}_{\text{ijk}}$: measurement *k* of the variable *j* in experiment *iy*_*ijk*_: model predicted value *k* of the variable *j* in experiment *i*

The Akaike information criterion (AIC) [25] for a given model is a function of the maximized log-likelihood (Eq. 1) and the number of estimated parameters (*N*_*p*_):

(2) $$\text{AIC}=-2J_{\text{ml}}(\text{p})+2N_{p}$$

Many functions have been suggested to compare two or more models. Despite the fact that several authors have questioned whether AIC is biased towards complex model structures [26], this function has been widely used as a metric to select the most adequate among hierarchical or nested models, since it encompasses model performance and model complexity [27]. It allows establishing a ranking of the models where the most adequate is the one with the smallest value of the criterion [25]. However, most of the available techniques for model selection based on the AIC, require the previous fitting of all the candidate models. Therefore, when the number of possible models is large or the simulation of the models is computationally expensive, these methodologies can become practically impossible [27].

In order to reduce the computational burden, in this work we used the AIC as cost function for finding the optimal set of parameters formed by a subset of binary parameters defining the model structure (e.g. presence or absence of a certain feedback loop) and another subset of integer and real parameters characterizing the model dynamics.

### Formulation of the MINLP

The formulation of the simultaneous model selection and identification problem is stated as an MINLP optimization problem. In mathematical terms, the general MINLP is defined as finding the vector of *n*_*r*_ continuous variables **p** and the vector of *n*_*i*_ integer variables **q** which minimize a scalar function *J*

(3) $$\underset{\text{p},\text{q}}{\min}\;J(\dot{y},y,p,q)$$

subject to: 

•System dynamics in the form of DAEs, with state variables **y**

(4) $$\begin{array}{c} f(\dot{y},y,p,q)=0\; \end{array}$$

(5) $$\begin{array}{c} \;y(t_{0})=y_{0}\; \end{array}$$

• Additional requirements in the form of equality and/or inequality constraints

(6) $$\begin{array}{ll} h(y,p,q)=0 & \; \end{array}$$

(7) $$\begin{array}{ll} g(y,p,q)\leq 0 & \; \end{array}$$

• Upper and lower bounds (superscripts U and L respectively) on decision variables

(8) $$\begin{array}{ll} p^{L}\leq p\leq p^{U} & \; \end{array}$$

(9) $$\begin{array}{ll} q^{L}\leq q\leq q^{U} & \; \end{array}$$

This set of constraints defines the feasible space *S*, while the feasible objective space *o* is the set *J* (**p**, **q**) | (**p**, **q**) ∈ *S*.

### Solution of the MINLP problem

The problem of parameter estimation is a crucial step in the development of models of biological systems [17]. Due to the nonlinear and dynamic nature of these systems and the usually sparse and noisy nature of the experimental data available, the resulting parameter estimation problem is frequently ill-conditioned and multi-modal. Therefore, traditional local methods may fail and there is a need to use more sophisticated techniques as global optimization (GO) to successfully fit the model. In our group, different efforts have been devoted to design metaheuristics for efficient and robust parameter estimation in biological models [28,29].

In the case of the MINLP problem at hand, the need to use GO methods is increased by the additional non-linearities coming from the binary and integer variables and the augmented size of the problem. ACOmi (Ant Colony Optization for mixed-integer problems) [30] and fSSm [31] are robust extensions of metaheuristics (Ant Colony optimization and Scatter Search, respectively) that enable the handling of mixed-integer variable search domains; therefore, they are ideal for solving the MINLP problem introduced in this work.

#### ***ACOmi***

ACOmi (Ant Colony Optization for mixed integer problems) is an extension of the ant colony optimization metaheuristic that enables to handle mixed integer variable search domains. In this method a new penalization strategy was introduced in order to extend the ACO framework to face constrained optimization problems. A detailed explanation of the hybrid implementation ACOmi, incorporating the extended ACO framework and a robust oracle penalty method, is given by [30].

#### ***fSSm***

fSSm is a new evolutionary method for complex-process optimization. It is partially based on the principles of the scatter search methodology, but making use of innovative strategies to be more effective in the context of complex-process optimization using a small number of tuning parameters. In particular, this method uses a new combination method based on path relinking, which considers a broader area around the population members than previous combination methods. It also uses a population-update method which improves the balance between intensification and diversification, as described in [31]. Although fSSm is mainly designed for continuous problems, a rounding operator has been implemented for handling integer and binary variables.

#### ***MISQP***

MISQP is a modified sequential quadratic programming method for solving MINLP problems. MISQP assumes that the model functions are smooth in the sense that an increment of an integer variable by one leads to a small change of function values but it does not require that the mixed-integer program is convex or relaxable (i.e. function values are evaluated only at integer points). Thus, this algorithm is expected to be more efficient than any other method that starts from a solution of the relaxed problem [32].

Moreover, in contrast to other local optimization solvers, the evaluation of the exact gradient is not always required for a proper convergence of SQP methods. The evaluation of the performance of the method used in this study, MISQP, on a test set of 186 academic test examples published in [33] showed that analytical partial derivatives subject to the integer variables do not improve robustness or efficiency, and the number of iterations is enlarged [34]. Diehl et al. [35] presented another SQP algorithm which does not require the evaluation of the exact constraint Jacobian matrix.

### Model identifiability, sensitivity and correlation analysis

Several powerful approaches have been recently developed to asses the identifiability of model parameters in systems biology models, namely, those exploiting the profile likelihood [36], Bayesian approaches using Markov Chain Monte Carlo (MCMC) [37], core-prediction analysis based on spread-searching optimization algorithms [38], and pseudo-global identifiability analysis using a Bayesian framework [39]. All these approaches aim to assess the quality of the estimated parameters by checking the practical identifiability of the model. This study aims to answer the following question: given a model structure, could the parameters of the model be uniquely identified from the available (limited and noisy) data [40,41]? The classical definition of identifiability requires the calculation of the rank of the Fisher Information Matrix (FIM) given by:

(10) $$\text{FIM}=\overset{\text{NM}}{\underset{i=1}{\sum }}\frac{1}{\sigma_{i}^{2}}{\left[\frac{\partial y_{i}}{\partial p}\right]}^{T}\left[\frac{\partial y_{i}}{\partial p}\right]$$

If the FIM is full rank the parameters are considered identifiable [42]. The parameters of a model are not identifiable when an infinite number of parameter sets fitting the experimental data with the same accuracy exist and the confidence intervals are infinite. Moreover, it is also interesting to study the parameter sensitivity and the correlation among parameters.

#### ***Sensitivity analysis***

Sensitivity analysis measures the importance of the parameters with respect to the influence of their variations on model predictions. The most widely used method is the local sensitivity analysis which consists of calculating the partial derivatives of the model state variables to the model parameters evaluated at the normal operating point where all the parameters have their nominal value. This method gives a linear approximation of how much a variable changes due to a given change in a parameter. The use of relative measures, where the sensitivity function is normalized by the value of the parameter and the state, is recommended to make these measures comparable for parameters and states of different order of magnitude:

(11) $$S_{\theta ,j}=\frac{p_{\theta }}{y_{i}}\frac{\delta y_{i}}{\delta p_{\theta }}$$

To lump the sensitivity of a parameter with respect to different states at different time points and different experiments, Brun et al. [43] recommend the use of the measure $\sigma_{\theta }^{\text{msqr}}$ as a ranking criterion in the context of weighted least squares estimation:

(12) $$\sigma_{\theta }^{\text{msqr}}=\sqrt{\overset{\text{NE}}{\underset{i=1}{\sum }}\overset{\text{NVi}}{\underset{j=1}{\sum }}\overset{{\text{NM}}_{\text{ij}}}{\underset{k=1}{\sum }}S_{\theta ,\text{ijk}}^{2}}$$

A high value of the sensitivity index means that a change in parameter *p*_*θ*_ has an important effect on the model outcome making the parameter *p*_*θ*_ identifiable with the data available if all the other parameters are fixed. Unless a parameter is unidentifiable due to total correlation with another parameter, the higher the sensitivity the more accurately the parameter can be identified and, on the other hand, a parameter with a small sensitivity will be very difficult to identify since any change on its value will have almost no influence on the model dynamics. Therefore, values of critical parameters should be thoroughly identified while parameters having a little effect can be simplified or even ignored [44].

The main drawback of local sensitivity indices is that they are computed at the nominal values used for the parameters and the behavior of the response function is described only locally in the parameter space. Moreover, preliminary experiments and parameter estimation tests should be carried out in order to obtain a first guess for the parameter values and an iterative scheme involving both steps is required to study the model sensitivity. In addition, these methods are linear; thus, they are not sufficient for dealing with complex models, especially those in which there are nonlinear interactions between parameters.

In contrast, global sensitivity analysis (GSA) methods evaluate the effect of a parameter while all other parameters are varied simultaneously, accounting for interactions between parameters without depending on the stipulation of a nominal point. In this work, a pseudo-global sensitivity analysis as described in [39] was used. For that, 2^10^ sampling points were generated in the parameter space by means of Sobol’ low-discrepancy sequences that guarantee an uniform distribution avoiding clustering and empty areas [45]. Then, Bayesian Derivative based Global Sensitivity Measures [39] were computed using SensSB toolbox [46] and their metrics were used to establish an importance ranking for the parameters.

#### ***Correlation analysis***

For models with several parameters, high parameter sensitivity, although necessary, does not ensure the identifiability of the model. In addition, the sensitivity functions of the parameters have to be linearly independent so a change in one parameter can not be compensated by changes in the other parameters. When the parameters are identifiable, we can study the degree of linear dependence among the sensitivity functions by means of a correlation analysis based on the Fisher Information Matrix (FIM) as described in [28]. This method requires the inversion of the FIM so it can only be applied when the parameters are identifiable and the FIM full rank. However, correlations among parameters close to +1 or -1 mean that the parameters are difficult to identify and the confidence intervals very large (although not infinite as in the case of nonidentifiable parameters). In that case, the model should be reduced by fixing some of the parameters to their nominal values or by properly grouping some sets.

In order to eliminate the dependence on a nominal point, a pseudo-global identifiability analysis as described in [39,46] was used. A correlation matrix was computed for each set of parameters used for the sensitivity analysis and a weighted average was obtained based on the maximum likelihood of each of the parameter sets. In this way, the influence of parameters not-likely to fit the data is minimized while the dependence on a nominal point is avoided.

### Dynamic model of the KdpD/KdpE system of *E*scherichia coli

Bacteria constantly monitor their environment and adapt immediately to changing conditions to survive. There are several adaptation mechanisms notably special signal transduction systems. A sensor kinase (*KdpD*) and a response regulator (*KdpE*) regulate the expression of the *KdpFABC* operon encoding the high affinity *K*^+^ uptake system of *Escherichia coli*. In [47], a mathematical model for the *KdpD/KdpE* two-component system was developed and calibrated with the available *in vitro* and *in vivo* experimental data. The model can be separated into two submodels connected in a unidirectional way. The parameters corresponding to the signal transduction part were estimated from *in vitro* data while the parameters of the gene expression functional unit were identified from *mRNA* and *KdpFABC* concentrations determined *in vivo* using various extracellular stimulus, $S=\frac{K^{+}}{K_{0}^{+}}$.

The dynamic model presented by Kremling and coworkers [47] (Model I) consists of 8 DAEs (6 ODEs and 2 AEs) and has 21 rate constants that were estimated from experimental data or fixed to literature values (Eq: 13-20):

(13) $$\begin{array}{lll} \;\frac{\text{dmRNA}}{\text{dt}} & =k_{\text{tr}}\;\left(\frac{{\text{DNA}}_{f}}{K\;{\text{DNA}}_{0}}\left(1\;+\;\frac{{\left({\text{KdpE}}_{f}^{p}\right)}^{2}}{\alpha \;K_{a}}\right)\right)\; & \\ & \;\times \;{\text{DNA}}_{0}\;-\;\left(k_{z}+\mu \right)\text{mRNA}\; \end{array}$$

(14) $$\begin{array}{ll} \;\frac{{\text{dKdpD}}_{0}}{\text{dt}} & =k_{\text{tl}}\;\text{mRNA}\;-\;\left(k_{d}+\mu \right){\text{KdpD}}_{0}\; \end{array}$$

(15) $$\begin{array}{lll} \;\frac{\text{dKdp}D^{P}}{\text{dt}} & =\left(k_{-2}\;\text{Kdp}E^{P}\;+\;k_{1}\right)\;\left({\text{KdpD}}_{0}-\text{Kdp}D^{P}\right)\;\; & \\ & \;-\;\left(k_{d}+k_{-1}+k_{2}\;\left({\text{KdpE}}_{0}-\text{Kdp}E^{P}\right)\right)\;\text{Kdp}D^{P}\; \end{array}$$

(16) $$\begin{array}{ll} \;\frac{{\text{dKdpE}}_{0}}{\text{dt}} & =k_{\text{tl}2}\;\text{mRNA}\;-\;\left(k_{d}+\mu \right){\text{KdpE}}_{0}\; \end{array}$$

(17) $$\begin{array}{lll} \;\frac{\text{dKdp}E^{P}}{\text{dt}} & =k_{2}\;\text{Kdp}D^{P}\;\left({\text{KdpE}}_{0}-\text{Kdp}E^{P}\right)\; & \\ & \;-\left(k_{d}+\left(k_{3f}+k_{-2}\right)\;\text{KdpD}\right)\text{Kdp}E^{P}\; \end{array}$$

(18) $$\begin{array}{ll} \;\frac{\text{dKdpFABC}}{\text{dt}} & =k_{\text{tl}3}\;\text{mRNA}\;-\;\left(k_{d2}+\mu \right)\text{KdpFABC}\; \end{array}$$

(19) $$\begin{array}{ll} 0 & =\text{Kdp}E^{P}-{\text{KdpE}}_{f}^{p}-2\frac{{\left({\text{KdpE}}_{f}^{p}\right)}^{2}{\text{DNA}}_{f}}{K_{a}}\left(1+\frac{1}{\mathrm{\alpha K}}\right)\; \end{array}$$

(20) $$\begin{array}{lll} 0 & ={\text{DNA}}_{0}\;-\;{\text{DNA}}_{f}\;\left(1+\frac{1}{K}\right)\;-\;\frac{{\left({\text{KdpE}}_{f}^{p}\right)}^{2}\;{\text{DNA}}_{f}}{K_{a}}\; & \\ & \;\times \left(1+\frac{1}{\alpha \;K}\right)\; \end{array}$$

where *mRNA* represents the concentration of messenger RNA, *KdpD*_0_ the total concentration of the sensor kinase, *KdpD*^*P*^ the concentration of the phosphorylated *KdpD*, *KdpE*_0_ the total concentration of the response regulator, *KdpE*^*P*^ the concentration of the phosphorylated *KdpE*, *KdpFABC* the concentration of the protein complex and ${\text{KdpE}}_{f}^{P}$ the concentration of the unbound response regulator.

## Results and discussion

Computations were carried out using Matlab™ (Version 7.9.0, R2009b; The Mathworks, MA, USA) running on a dual INTEL®;XEON®;2.13 GHz CPU desktop under Windows 7. All the scripts needed to reproduce the results presented in the following are provided in the Additional file 1.

### Identifiability analysis of the original model

Simulation studies showed that the concentration of *KdpD*^*P*^ was very low and almost in steady state. Therefore, we removed equation (15) from the model and consequently we fixed the concentration of *KdpD*^*P*^ to its initial value and parameters *k*_1_ and *k*_-1_ were eliminated.

A local identifiability analysis of the original model with the best set of parameters was performed. As already suggested by Kremling *et al*[47], the full set of parameters is not uniquely identifiable with the available *in vivo* data; thus, some of the parameters were fixed to literature values or to values obtained from *in vitro* data.

The importance ranking of the parameters estimated from the *in vivo* data revealed that parameter *k*_-2_ has the lowest sensitivity index (it accounts for the 0.002% of the total model sensitivity) while the two most relevant parameters, *k*_*tr*_ and *DNA*_0_ represent 75% of the total sensitivity. Hence, *k*_-2_ was fixed to its nominal value and special attention was payed to the set of most influential parameters. The parameter *μ* presented high correlations with other parameters so it was fixed to its experimental value *μ* = 0.5 *l*/*h*. Other pairs of parameters showed also high correlation among them but they could still be identified.

These modifications led to a second formulation of the model (Model II) with 7 DAEs and 17 parameters that fits the experimental data equally well.

### New hypotheses for the *KdpD/KdpE* two component system

**Figure 2:**
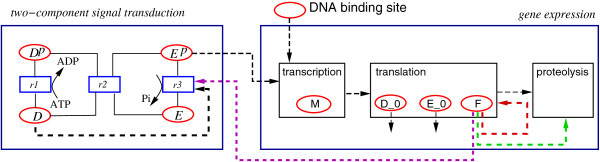
**Scheme of the reaction mechanism for the*KdpD/KdpE* two component system.***KdpD/KdpE* model with the feedback loops: regulation of translation (red), regulation of proteolysis (green), and a modified stimulus counteraction (purple).

Based on unpublished data of a mutant strain with impaired *K*^+^ uptake properties, the existence of two new feedback loops concerning the regulation of translation and the regulation of proteolysis could be derived from the observations (see Figure 2). Moreover, a different expression for the existing stimulus counteraction feedback loop was postulated. The selection between competing models is done by using three binary parameters: *bin*_1_ and *bin*_2_, which take values 0 or 1 depending on the absence or presence of feedback loops, and *bin*_3_, which determines the function representing the stimulus counteraction. The feedback loops were modeled using S-shaped functions similar to the Hill equation where the Hill coefficient (*n*) represents the number of ligand molecules that are required to bind to a receptor to produce a functional effect. Typically, these functions are moderately steep; hence, the different exponents were allowed to take integer values between 0 and 3 [48-50]. Therefore, equation (18) was modified according to:

(21) $$\frac{\text{dKdpFABC}}{\text{dt}}=k_{\text{tl}3}\;\text{mRNA}\;R_{1}\;-\;(k_{d2}\;R_{2}+\mu )\text{KdpFABC}$$

where

• Regulation of translation (*R*_1_)

(22) $$R_{1}=\left\{\begin{array}{cc} 1, & \text{if}\;{\text{bin}}_{1}=0\\ \frac{1}{\text{KdpFAB}C^{n_{1}}\;+\;k_{\text{trans}}}, & \text{if}\;{\text{bin}}_{1}=1 \end{array}\right)$$

• Regulation of proteolysis (*R*_2_)

(23) $$R_{2}=\left\{\begin{array}{cc} 1, & \text{if}\;{\text{bin}}_{2}=0\\ \frac{\text{KdpFAB}C^{n_{2}}}{\text{KdpFAB}C^{n_{3}}\;+\;k_{\text{deg}}}, & \text{if}\;{\text{bin}}_{2}=1 \end{array}\right)$$

• Stimulus counteraction (*R*_3_)

In order to account for the different *K*^+^ uptake properties of the two strains, the model is simulated twice considering different expressions for *k*_3*f*_ (existence and lack of feedback loop for the wild and mutant strains, respectively). In the case of the mutant strain, *k*_3*f*_ is given by:

(24) $$k_{3f}=k_{3}\;\frac{K^{+}}{K_{0}^{+}}$$

While two different expressions were hypothesized for the wild strain:

(25) $$k_{3f}=R_{3}=\left\{\begin{array}{cc} k_{3}\;\frac{K^{+}}{K_{0}^{+}}\;k_{\text{hy}}\;\text{KdpFAB}C^{n_{4}}, & \;\text{if}\;{\text{bin}}_{3}=0\\ k_{3}\;\frac{K^{+}}{K_{0}^{+}}\;k_{\text{hy}}\;\frac{\text{KdpFAB}C^{n_{4}}}{\text{KdpFAB}C^{n_{5}}\;+\;K_{\text{hy}}}, & \;\text{if}\;{\text{bin}}_{3}=1 \end{array}\right)$$

Note that the dynamics of the mutant strain do not depend on parameters *bin*_3_, *n*_4_, *n*_5_, *k*_*hy*_, and *k*_*hy*_.

These possible new loops were integrated with the Model II considering a superstructure, which has a total of 25 degrees of freedom: 17 reals, 5 integers and 3 binaries, resulting in 1700 nested models. In a traditional setting, each of this model should be identified (calibrated) from experimental data by solving the corresponding minimization problem, that is, a nonlinear-programming problem subject to differential-algebraic constraints (NLP-DAEs), prior model selection. Since the solution of each problem is quite computationally expensive, this is obviously not tractable. As an alternative, we applied the strategy outlined above and performed a simultaneous selection and identification via MINLP optimization.

### MINLP solutions

In order to illustrate the capabilities of the methodology presented in this work, we generated *in silico* data *via* simulation using a nominal set for parameters and a certain model structure. Starting from a known structure and known parameter values allows us to asses the performance of the MINLP formulation by checking if it is able to recover the original model.

Therefore, we generated *in silico* data for a wild and a mutant strain, defective in the uptake of *K*^+^ via the *KdpFABC* system, considering the parameters shown in Table 1 as nominal parameters. In this model, the regulation of translation and the regulation of proteolysis are active. Moreover, the stimulus counteractions presents linear dynamics with a kinetic order of three for *KdpFABC*. For each strain, five different values of *K*^+^ concentration were considered (1, 10, 50, 100 and 500 mM) and to create a more realistic scenario we considered that we can only measure *mRNA* and *KdpFABC* with an heteroscedastic error of 5%.

**Table 1:** Nominal value for the parameters and MINLP best solution

**Parameter**	**Nominal value**	**MINLP solution**
*k*_2_	5.18E+07	4.74E+07
*k*_3_	9.76E+01	1.20E+02
*α*	5.79E-02	5.23E-02
*k*_*tr*_	1.00E+03	8.66E+02
*k*_*tl*_	4.96E+03	4.28E+03
*k*_*tl*2_	1.03E+03	1.27E+03
*k*_*tl*3_	2.05E+03	1.64E+03
*k*_*z*_	4.99E+01	6.06E+01
*k*_*d*2_	1.00E+01	1.25E+01
*DNA*_0_	6.16E-04	7.08E-04
*K*_*a*_	1.82E-07	2.11E-07
*K*	1.00E+03	8.48E+02
*k*_*d*_	1.18E+00	9.55E-01
*k*_*hy*_	2.00E+06	1.76E+06
*k*_*transf*_	9.74E-01	8.07E-01
*k*_*degf*_	1.36E-01	1.84E-01
*k*_*hy*_	-	-
*n*_1_:	3	3
*n*_2_:	1	1
*n*_3_:	2	3
*n*_4_:	3	3
*n*_5_:	0	0
*bin*_1_:	1	1
*bin*_2_:	1	1
*bin*_3_:	0	0

The bounds for the real parameters were taken at 50% and 200% around the initial values and for the integers they are based on the typical values of Hill coefficients, from 0 to 3. The value of parameter *k*_*hy*_ is not reported because it is inactive when *bin*_3_ = 0.

**Figure 3:**
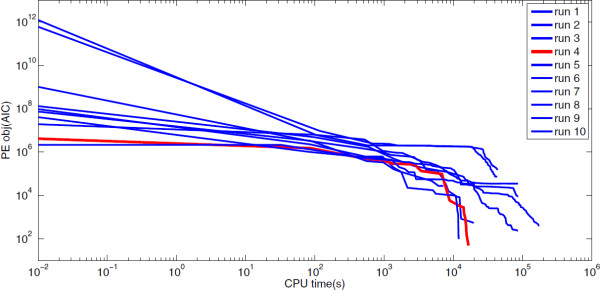
**Convergence curve of fSSm for the MINLP problem.** Convergence curve of fSSm (AIC value versus computational time, in seconds, using a PC/INTEL XEON CPU, 2.13 GHz).

Subsequently, we solved the MINLP problem using fSSm and ACOmi as optimization methods and the AIC as cost function. Both, fSSm and ACOmi, could solve the problem of simultaneous model selection and parameter identification in an acceptable computation time, while fSSm showed a better overall performance (data not shown). The convergence curves for ten runs of fSSm (AIC value versus computational time) are depicted in Figure 3 showing a fast convergence rate particularly at the initial stage of the optimization. The convergence curve of the run which achieved the best result is highlighted in red.

**Figure 4:**
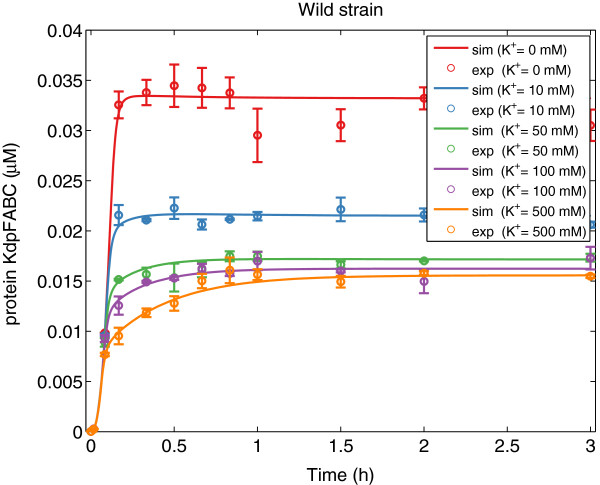
**KdpFABC data*versus* model prediction for the wild strain.** Predicted (solid lines) and experimental *in silico* (markers) behavior for the protein *KdpFABC* for the wild strain at different concentrations of *K*^+^ using Model III with the best parameter set.

**Figure 5:**
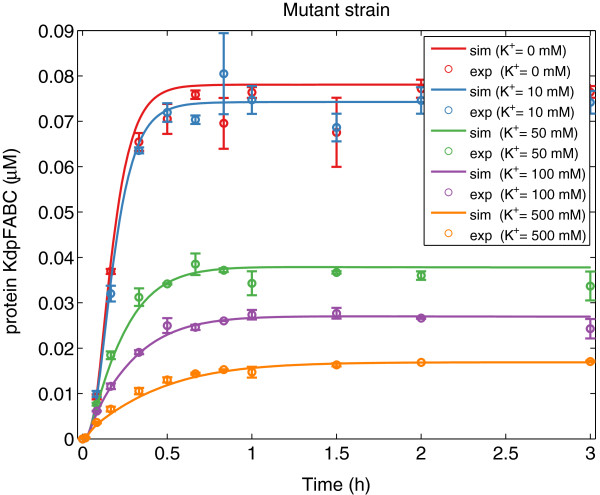
**KdpFABC data*versus* model prediction for the mutant strain.** Predicted (solid lines) and experimental *in silico* (markers) behavior for the protein *KdpFABC* for the mutant strain at different concentrations of *K*^+^ using Model III with the best parameter set.

**Figure 6:**
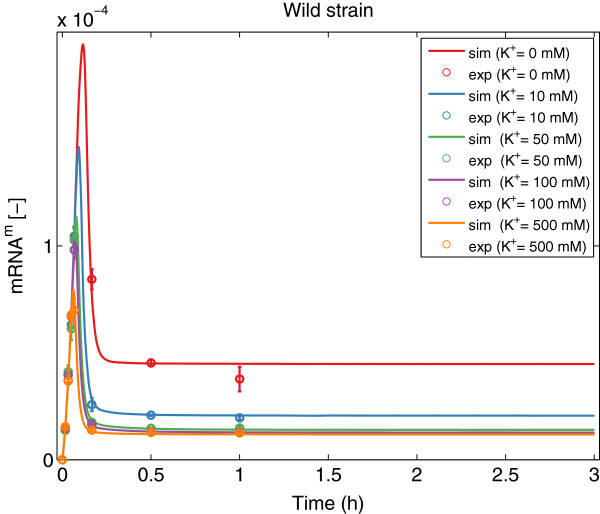
**mRNA data*versus* model prediction for the wild strain.** Predicted (solid lines) and experimental *in silico* (markers) behavior for the *mRNA* for the wild strain at different concentrations of *K*^+^ using Model III with the best parameter set.

**Figure 7:**
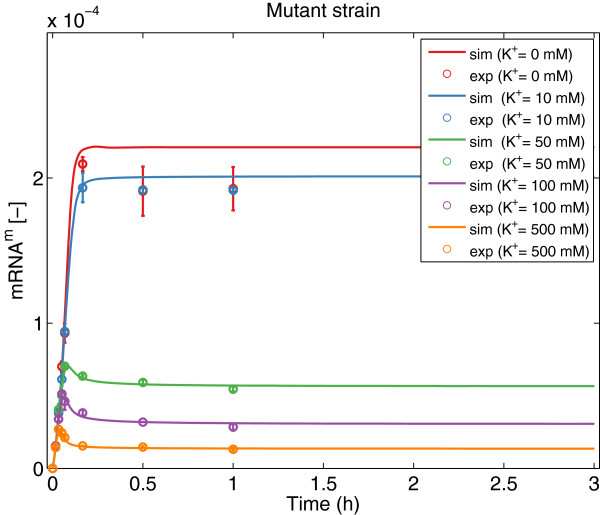
**mRNA data*versus* model prediction for the mutant strain.** Predicted (solid lines) and experimental *in silico* (markers) behavior for the *mRNA* for the mutant strain at different concentrations of *K*^+^ using Model III with the best parameter set.

As can be seen in Table 1, fSSm was able to recover the model structure used to generate the *in silico* data (the same loops are active) and the optimal parameters differ from the nominal values less than 20%. Figures 4, 5, 6, 7 show a good agreement between the new model (Model III) and the *in silico* data. The mean of the residuals is 4.4%, very close to the experimental error. Although not every realization reached the same value of the cost function, the regulation of translation and regulation of proteolysis are active for the 10 solutions (*bin*_1_ = *bin*_2_ = 1). For some of the realizations, *bin*_3_ is equal to 1 indicating that the two different expressions for the wild strain are difficult to distinguish with the available data. Moreover, the proposed method allows formulating biological hypotheses in a much more compact way and this example -although using *in silico* data- shows that also complex systems can be handled.

### Checking the multi-modality of the MINLP

**Figure 8:**
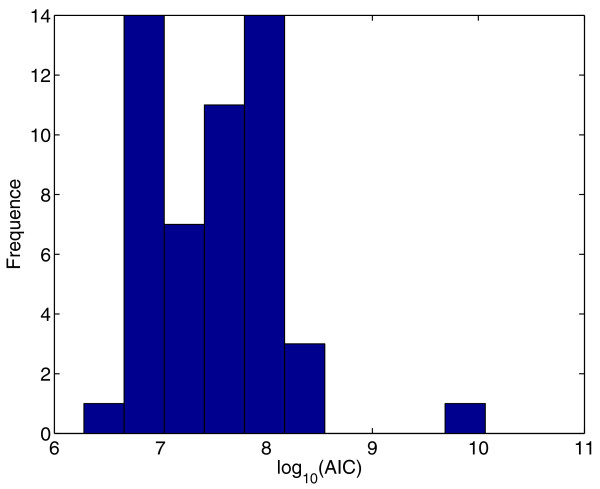
**Multistart of the local Solver MISQP on the MINLP problem.** Histogram of a multi-start of 50 runs using the local solver *MISQP*.

In order to assess the multi-modality of the MINLP problem, a traditional multi-start approach (i.e. choosing a large set of random initial points from inside the parameter bounds, and performing local searchers from each one) using MISQP was performed. The histogram in Figure 8 represents the frequency of the solutions for a multi-start of 50 runs showing that all the solutions obtained are local solutions very far from the global optimum (three orders of magnitude higher than the global optimum).

### Identifiability analysis of the resulting model

The FIM computed for the best set of parameters obtained by the global solver is full rank; therefore, we can assert that the parameters are locally identifiable.

**Figure 9:**
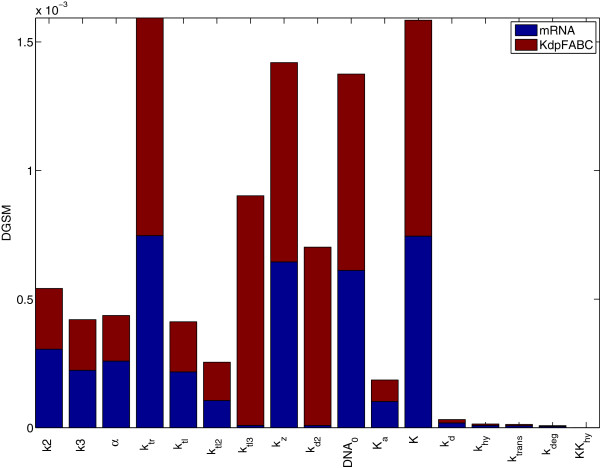
**Pseudo-global sensitivity for Model III.** Pseudo-global sensitivity of Model III with respect to the two measured states (protein *KdpFABC* and *mRNA*).

**Figure 10:**
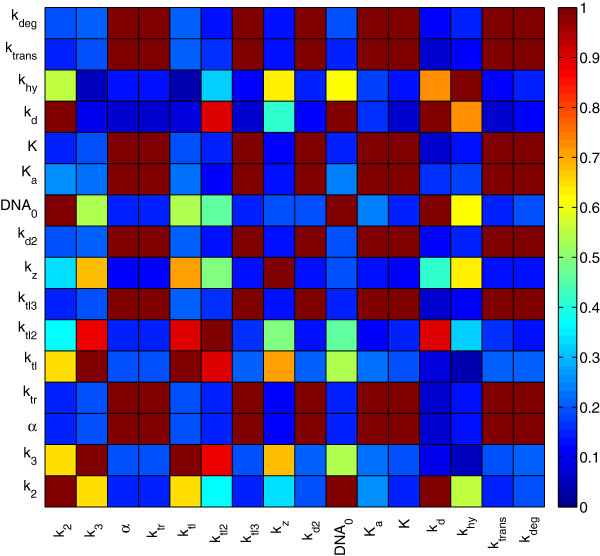
**Correlation matrix for Model III.** Correlation matrix for Model III with the best parameter set.

Figure 9 represents the sensitivity of the two measured variables, *mRNA* and protein complex *KdpFABC*, with respect to the 17 real parameters of Model III. The pseudo-global sensitivity analysis revealed that the model dynamics are very sensitive to parameters *DNA*_0_ and *k*_*tr*_, in agreement with the results of the local sensitivity analysis for Model II. Moreover, the concentration of both *mRNA* and *KdpFABC* showed high sensitivity to parameters *K* and *k*_*z*_. Since the stimulus counteraction appears to be linear (*bin*_3_ = 0), parameter *k*_*hy*_ is not playing a role in the model, therefore its sensitivity index is zero. For this reason, we have excluded it from the correlation matrix computation represented in Figure 10.

The correlation matrix shows several pairs of parameters highly correlated what explains the difficulties encountered by the local method in finding the global solution. Despite the identifiability difficulties of this problem, which make most of the solvers fail when trying to solve it, the residuals for the solution obtained by fSSm are small indicating a precise parameter estimation, *e.g.*, the estimated values are close to the experimental data.

### Methodology strengths and limitations

The goal of this study is not to solve the general problem of model inference but a dense subcase of it, i.e., the discrimination among a subset of nested competing models and simultaneous estimation of the model parameters. In other words, we consider the very frequent situation in systems biology where a first model is available based on previous knowledge but new experimental information allow to formulate different hypotheses to refine it. Thus, instead of solving a general inference problem (i.e. find the model structure plus the parameters from a set of data), we consider a subproblem which is smaller (although still very challenging) and dense (so sparsity is not an issue), and which, therefore, does not suffer from many of the ill-posedness and ill-conditioning maladies of the general inference problem [51,52]. Despite the usefulness and broad applicability of the presented approach for model development in systems biology, there are three major limitations worth mentioning here: 

• Scaling up to large-scale models: the corresponding MINLPs might become rather large and therefore the computational effort needed for their solution might become prohibitive.

• Non-uniqueness of biochemical reaction mechanisms: it is known that biochemical reaction networks with different structure and/or parametrization may produce the same dynamic response describing the time-evolution of species concentrations (see the recent discussion and results in [52]) difficulting the solution of the associated MINLPs. Fortunately, and following our comments above, this work considers a dense subcase of the general inference problem, so these issues are not as important. In fact, our approach can be interpreted as the application of extra constraints that can be used to reduce uniqueness and identifiability issues.

• Model identification/selection metric: the use of more advanced metrics for model selection such as the likelihood ratio or the F-test can not be used in this approach since they rely on pair-wise comparisons. However, in the presented methodology the AIC could be replaced by any other metric for model selection as long as it can establish a ranking for the set of competing models encompassing model performance and model complexity.

## Conclusions

**Figure 11:**
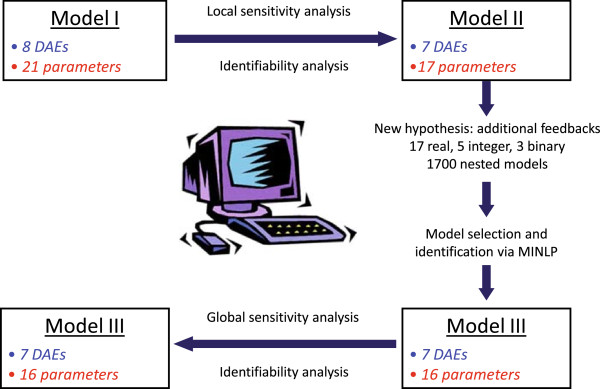
**Model selection scheme.** Model selection scheme: Local sensitivity analysis and identifiability analysis allowed to reduce Model I leading to Model II. Subsequently, new hypotheses and model selection and identification via MINLP were conducted to formulate Model III. The identifiability of Model III was assessed by means of a pseudo-global sensitivity approach and correlation analysis indicating that no further modifications were required.

Here we have considered the model-building cycle where an initial model, based on existing data and *a priori* knowledge of the system, is subsequently refined by hypotheses-driven iterations (see Figure 11).

We consider this cycle in a more compact way grouping sets of hypotheses together and formulating a parameterized set of nested models, from which the best alternative must be selected. We then formulate the decision problem as an MINLP-based optimization for simultaneous model selection and parameter identification.

This procedure has been applied to a case study considering potassium homeostasis in bacteria, arriving to the following conclusions: (i) the presented MINLP-based approach for nested-model selection is a powerful methodology for model selection and identification in systems biology; and (ii) for the case study considered, it has resulted in a model that presents a better fit to the *in silico* generated experimental data.

## Competing interests

The authors declare that they have no competing interests.

## Authors’ contributions

MRF and MR implemented the model options and performed the analysis of the novel methodology, carrying out the necessary computations. MRF performed the analysis of the optimization results, the identifiability computations and assisted in the coordination of the study. JRB and AK conceived of the study and participated in its design and coordination. MR, JRB and MRF drafted the manuscript. All authors read and approved the final manuscript.

## Supplementary Material

Additional file 1 **K_homeostasis_MINLP.** K_homeostasis_MINLP.zip contains all the scripts needed to reproduce the results presented in this manuscript using the toolbox SensSB [46]. SensSB toolbox and related documentation can be downloaded from the following web site: http://www.iim.csic.es/~gingproc/SensSB.html Click here for file
